# Differences in plasma metabolome between non-Hispanic White and non-Hispanic Black women

**DOI:** 10.1186/s12916-025-03988-1

**Published:** 2025-03-13

**Authors:** Ghazaleh Pourali, Liang Li, Myung Sik Jeon, Jingqin Luo, Chongliang Luo, Adetunji T. Toriola

**Affiliations:** 1 Division of Public Health Sciences, Department of Surgery, Washington University School of Medicine, St. Louis, MO USA; 2 Siteman Cancer Center Biostatistics Shared Resource, Division of Public Health Sciences, Department of Surgery, Washington University School of Medicine, St. Louis, MO USA; 3 Siteman Cancer Center, Washington University School of Medicine, St. Louis, MO USA

**Keywords:** Metabolomics, Metabolites, Non-Hispanic Black, Non-Hispanic White, Body mass index, Women, Xenobiotics

## Abstract

**Background:**

To understand potential racial differences in disease susceptibility and develop targeted prevention strategies, it is essential to establish biological differences between racial groups in healthy individuals. However, knowledge about how race impacts metabolites is limited. We therefore performed a cross-sectional study using comprehensive metabolomics analysis to investigate racial differences in metabolites among 506 non-Hispanic White (NHW) women and 163 non-Hispanic Black (NHB) women.

**Methods:**

We performed untargeted plasma metabolomic profiling using Metabolon’s platform (Durham, NC®) and identified 1074 metabolites in 9 super-pathways. We used multivariable linear regression models, adjusted for confounders, to identify associations between race and metabolites. We applied a Bonferroni correction (*p*-value < 10^–5^) to account for multiple testing.

**Results:**

We identified 26 metabolites that differed significantly between NHW and NHB women. Seven, 10, 17, and 23 metabolites showed absolute percentage differences ≥ 50, ≥ 40%, ≥ 30%, and ≥ 20%, respectively. Xenobiotics (*n* = 5) and amino acids (*n* = 2) exhibited the largest absolute percentage differences (≥ 50%) between NHB and NHW women. In the xenobiotics super-pathway, NHB women had higher thymol sulfate, 2-naphthol sulfate, and 2-hydroxyfluorene sulfate, derived from the exposure to polycyclic aromatic hydrocarbons, while NHW women had higher xanthine metabolites. In the amino acid super-pathway, lysine and tryptophan metabolites were lower in NHB women.

**Conclusions:**

We report differences in several metabolites between NHW and NHB women. These findings require validation in a different study and could provide insight into investigating how racial differences in metabolites may impact disease burden across diverse populations.

**Supplementary Information:**

The online version contains supplementary material available at  10.1186/s12916-025-03988-1 .

## Background

Racial differences in health outcomes are well-documented, with non-Hispanic Black populations (NHBs) experiencing disproportionately higher rates of chronic diseases, including metabolic disorders like obesity, diabetes, and cardiovascular disease compared with non-Hispanic White populations (NHWs) [1, 2]. On the other hand, NHWs have higher lifetime rates of mood and anxiety disorders [3]. While these differences in disease prevalence and outcomes are widely recognized, the underlying biological mechanisms remain poorly understood, particularly in healthy individuals. To effectively address these health differences and develop targeted prevention strategies, it is crucial to establish whether baseline biological differences exist between racial groups.

One promising approach to uncover these biological differences is through the study of metabolites. Metabolites are key players in cellular functions and have been implicated in diseases including metabolic syndrome, diabetes, and cancer [4]. Metabolomics, the comprehensive study of these molecules, has emerged as a powerful tool for evaluating the body's metabolic state [5]. Metabolite profiles can directly reflect the phenotype, offering insights into physiological states [6]. They can also serve as early biomarkers of disease, potentially years before clinical symptoms appear [7]. Moreover, metabolites represent the downstream effects of both genetic and environmental factors, which makes them valuable to understand complex biological processes and potential disease etiologies [8].

While previous research has established associations between metabolites and factors such as age and sex [9, 10], the associations of race with the metabolome remain understudied. Many metabolomic studies have been conducted primarily in NHW populations, limiting our understanding of metabolic profiles in other racial and ethnic groups. Additionally, many studies have focused on disease states [11, 12], with less attention given to understanding racial differences in metabolomic profiles in healthy individuals. Some studies have had limited sample sizes, particularly for NHB participants [13], limiting robust statistical analysis. Furthermore, the use of targeted metabolomics approaches in some studies may have missed important racial differences in unexplored metabolic pathways. To our knowledge, only a limited number of studies have investigated racial differences in a wide range of plasma metabolites [14, 15]. For instance, in the Women's Health Initiative-Observational Study, 152 metabolites were identified as significantly different between NHB and NHW women. These differences were particularly in lipid metabolites and amino acids. However, this study analyzed only 472 metabolites [14]. Another study performed metabolomics analysis in a cohort of 175 women and 175 men, including 171 NHB and 179 NHW, but was limited by its relatively small sample size [15].

Our study aims to address these limitations by investigating plasma metabolomic profiles in women using an untargeted metabolomics approach, which allows us to investigate previously unexamined metabolic pathways. With a large sample size and an untargeted approach, study findings should provide a robust analysis of racial differences in metabolites.

## Methods

### Study population

This was a cross-sectional study focused on 163 NHB and 506 NHW women recruited during annual screening mammograms at Washington University School of Medicine, St. Louis, MO. Women were recruited in two phases from December 2015 to October 2016 and September 2020 to February 2022. Detailed population characteristics have been previously published [16]. In summary, inclusion criteria required participants to be premenopausal, non-pregnant, without breast augmentation, without a history of cancer, and not using selective estrogen receptor modulators in the past six months [16]. All participants provided written informed consent, and the study adhered to the Declaration of Helsinki, with approval from the Washington University Institutional Review Board. Participants completed comprehensive questionnaires, which included self-reported race and underwent physical measurements during their mammography visit. Fasting plasma samples were collected and rapidly stored at -80°C [16].

### Metabolomic profiling

Plasma samples were analyzed by Metabolon (Durham, NC®) using their ultra-high-performance liquid chromatography–mass spectrometry (UHPLC–MS) technology. Metabolite identification involved comparing spectral peaks to a reference library of chemical standards, with visual inspection for quality control [17]. This analysis identified 1074 metabolites across 9 super-pathways including amino acid, carbohydrate, lipid, cofactors and vitamins, energy, nucleotide, peptide, partially characterized molecules, and xenobiotics. Metabolite quantification involved normalizing signal intensities against internal standards and factoring in the amount of standard added [18]. Quality control included standard reference samples in the analysis runs and assessment of median relative standard deviation (RSD) between replicates, which was 8%. To account for potential variations between batches, raw metabolite readings were normalized by dividing each metabolite's value by its median value across all samples in the same batch.

### Statistical analysis

Analyses used ComBat-normalized values to account for batch effects. We excluded 246 metabolites with excessive missing values (≥ 300 women), corresponding to metabolites with more than 45% missing data. We used this threshold to exclude those metabolites that have too many missing values (> 45%). This number is consistent with our previous publications [19] and also follows the common “50% rule”, i.e. exclude those metabolites that have more than 50% missing values [20]. Among the included metabolites, the proportion of missing data ranged from 0 to 45%, with an average of 7% (Q1 = 0%, Q2 = 0.4%, Q3 = 9.7%). The remaining missing values were imputed using the 10-nearest neighbor method [21], which calculates a Euclidean distance metric to identify the 10 closest observations and then averages their values to estimate the missing data [22]. This global imputation approach, compared to the approaches that only rely on a subset of metabolites, obtains better imputation of the missing values of the analyzed metabolites as it incorporates the dependence between metabolites [23]. We performed Spearman correlation tests to assess the associations between metabolites and body mass index (BMI). We investigated the associations of race (primary exposure) and metabolites (outcomes) using multivariable linear regression models, adjusting for age (continuous), BMI (continuous), alcohol consumption (current use: yes, no), and education level (high school or less, post-high school/some college, college graduate, and postgraduate). In these models, variables such as oral contraceptive use (yes, no) and family history of breast cancer (yes, no) were initially considered but excluded due to minimal impact on metabolites to be considered as confounders. Metabolites were log-transformed for regression analyses to meet normality and homoscedasticity assumptions. Linear regression coefficients were subsequently back-transformed in the original scale as percentage differences. Additionally, sensitivity analyses were conducted in a subset of women without a family history of breast cancer (*N* = 516). Multiple testing was accounted for using Bonferroni correction in correlation and regression analyses. Pathway enrichment analyses were performed using a hypergeometric test for each super-pathway, comparing the number of significant metabolites in each super-pathway to the total number of metabolites in that super-pathway and the overall number of significant metabolites. Statistical significance was defined as a Bonferroni-adjusted *p*-value < 10^–5^ for both linear regression and correlation tests.

## Results

### Participant demographics

Among the 669 women, 506 (75.5%) were NHW and 163 (24.5%) were NHB (Table 1). Mean ages were similar for NHW (45.8 years) and NHB women (46.5 years). Mean BMI and body fat percentage were higher in NHB women (34.6 kg/m^2^, 46.3%) compared with NHW women (28.7 kg/m^2^, 38.9%). Alcohol consumption was more prevalent in NHW women (78.5%) compared with NHB women (47.2%). NHW women had higher education levels.

**Table 1:** Characteristics of 669 women recruited at the Joanne Knight breast health center, Washington University School of Medicine, St. Louis, MO

	Non-Hispanic White	Non-Hispanic Black	*P*-value^a^
Number	506	163	
Age at Enrollment, Mean (SD)	45.8 (4.6)	46.5 (4.1)	0.097
BMI, Mean (SD)	28.7 (6.7)	34.6 (7.7)	< 0.001
Body Fat Percentage, Mean (SD)	38.9 (10.4)	46.3 (11.1)	< 0.001
Family History of Breast Cancer, n (%)			
No	384 (75.9)	132 (81.0)	0.002
Yes	117 (23.1)	24 (14.7)	
Missing	5 (1.0)	7 (4.3)	
Oral Contraceptive Use, n (%)			
No	44 (8.7)	29 (17.8)	0.001
Yes	462 (91.3)	134 (82.2)	
Alcohol Consumption, n (%)			
No	109 (21.5)	86 (52.8)	< 0.001
Yes	397 (78.5)	77 (47.2)	
Education, n (%)			
High school or less	18 (3.6)	32 (19.6)	< 0.001
Post-high school/some college	63 (12.5)	41 (25.2)	
College Graduate	197 (38.9)	37 (22.7)	
Postgraduate	212 (41.9)	34 (20.9)	
Missing	16 (3.2)	19 (11.7)	

^a^Statistical significance was determined using the Wilcoxon rank-sum test for continuous variables and the Chi-square test for categorical variables

*Abbreviations*: *SD* standard deviation, *BMI* body mass index

### Correlations of metabolites with BMI

Of the 828 metabolites analyzed, 281 were significantly correlated with BMI at Bonferroni-adjusted *p*-value < 10^–5^. Metabolites within the lipid and amino acid super-pathways had the strongest positive correlations with BMI. The top three positive correlations with BMI were cortolone glucuronide (1) (*r* = 0.47, *p*-value = 8.9 × 10^–37^), hydroxyasparagine (*r* = 0.45, *p*-value = 6.3 × 10^–34^), and glutamate (*r* = 0.43, *p*-value = 6.4 × 10^–31^). Conversely, inverse correlations with BMI were observed in metabolites in cofactors and vitamins, xenobiotics, and lipid super-pathways. The top three inverse correlations with BMI were beta-cryptoxanthin (*r* = -0.44, *p*-value = 3.8 × 10^–32^), tartronate (hydroxymalonate) (*r* = -0.41, *p*-value = 7.2 × 10^–27^), and 3beta-hydroxy-5-cholestenoate (*r* = -0.40, *p*-value = 2.6 × 10^–25^) (Additional file 1: Table S1).

### Associations of race with metabolites

**Fig. 1:**
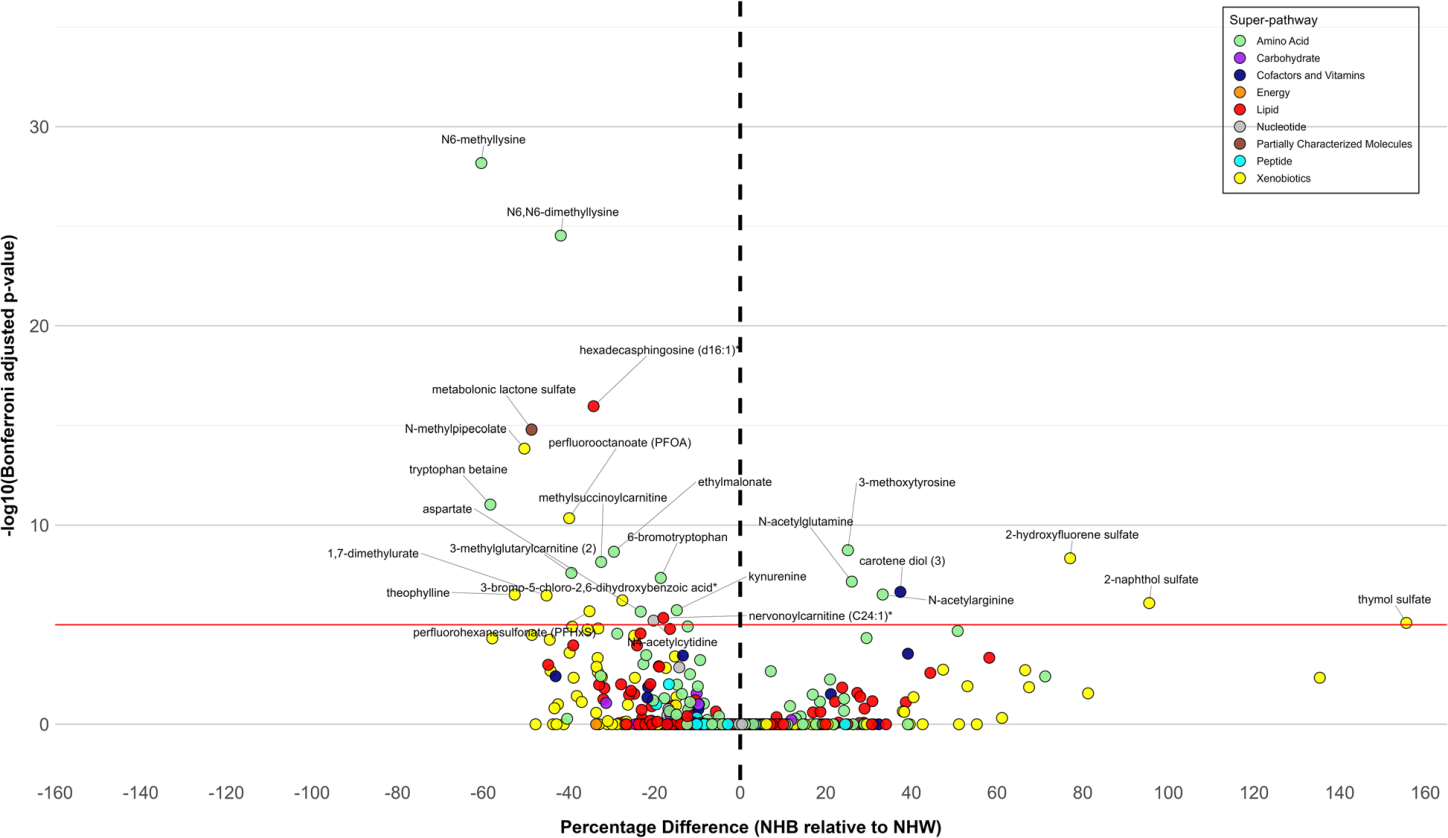
Associations of Race with Metabolites. Multivariable linear regression analysis was performed on the log-transformed metabolites and race (non-Hispanic White, non-Hispanic Black), adjusted for age, BMI at enrollment, alcohol consumption, and education. Multiple hypothesis testing was adjusted using Bonferroni correction. Percentage difference was back-transformed linear regression coefficients, calculated as ${100\times (e}^{\beta }-1)$. Name of metabolites with Bonferroni-adjusted *p*-value ≤${10}^{-5}$ were labeled. BMI, body mass index; NHB, non-Hispanic Black; NHW, non-Hispanic White

Our analysis revealed significant racial differences in metabolite profiles between NHB and NHW women. At the super-pathway level, we found significant enrichment in the amino acid (*p*-value = 0.02) and xenobiotics (*p*-value = 0.02) super-pathways (Table 2). At the metabolite level, we identified twenty-six metabolites (3.1% of the total) significantly associated with race at a Bonferroni-adjusted *p*-value < 10^–5^ (Fig. 1, Table 3). Of these, 19 metabolites were higher in NHW women, and 7 metabolites were higher in NHB women. These metabolites belong to amino acid (*n* = 12), xenobiotics (*n* = 9), lipid (*n* = 2), cofactors and vitamins (*n* = 1), nucleotide (*n* = 1), and partially characterized molecules (*n* = 1) super-pathways. All results, including those not reaching statistical significance, are presented in Additional file 1: Table S2.

**Table 2:** Super-pathways enriched with metabolites that differ between Non-Hispanic White and Non-Hispanic Black Women

Super-pathway^a^	Total Metabolites	Significant Metabolites	Bonferroni-adjusted *P*-value
Amino Acid	211	12	0.02
Cofactors and Vitamins	37	1	0.70
Lipid	305	2	1.00
Nucleotide	38	1	0.71
Partially Characterized Molecules	22	1	0.51
Xenobiotics	138	9	0.02

^a^Carbohydrate, peptide, and energy super-pathways have no metabolites statistically significant at 10^–5^ level

**Table 3:** Metabolites with significant differences between (Non-Hispanic Black and Non-Hispanic White Women^a, b^

Metabolite	Super-pathway	Sub-pathway	Percentage Difference (%)^c^	Bonferroni-adjusted *P*-value
*Higher in non-Hispanic White women*				
N6-methyllysine	Amino Acid	Lysine Metabolism	-60.5 (-65.9, -54.2)	6.7E-29
Tryptophan betaine	Amino Acid	Tryptophan Metabolism	-58.4 (-66.5, -48.3)	9.2E-12
Theophylline	Xenobiotics	Xanthine Metabolism	-52.7 (-62.4, -40.4)	3.1E-07
N-methyl pipecolate	Xenobiotics	Bacterial/Fungal	-50.4 (-57.6, -42.0)	1.4E-14
Metabolomic lactone sulfate	Partially Characterized Molecules	Partially Characterized Molecules	-48.7 (-55.7, -40.7)	1.6E-15
1,7-dimethylurate	Xenobiotics	Xanthine Metabolism	-45.2 (-54.5, -34.0)	3.4E-07
N6, N6-dimethyllysine	Amino Acid	Lysine Metabolism	-41.9 (-47.0, -36.3)	2.9E-25
Perfluorooctanoate (PFOA)	Xenobiotics	Chemical	-40.0 (-47.3, -31.6)	4.4E-11
3-methylglutarylcarnitine (2)	Amino Acid	Leucine, Isoleucine and Valine Metabolism	-39.4 (-47.6, -30.0)	2.5E-08
Perfluorohexanesulfonate (PFHxS)	Xenobiotics	Chemical	-35.2 (-43.7, -25.4)	2.1E-06
Hexadecasphingosine (d16:1)*	Lipid	Sphingosines	-34.2 (-39.8, -28.2)	1.1E-16
Methylsuccinoylcarnitine	Amino Acid	Leucine, Isoleucine and Valine Metabolism	-32.5 (-39.6, -24.6)	7.0E-09
Ethylmalonate	Amino Acid	Leucine, Isoleucine and Valine Metabolism	-29.5 (-36.0, -22.4)	2.1E-09
3-bromo-5-chloro-2,6-dihydroxybenzoic acid*	Xenobiotics	Chemical	-27.5 (-34.5, -19.8)	5.9E-07
Aspartate	Amino Acid	Alanine and Aspartate Metabolism	-23.2 (-29.6, -16.4)	2.2E-06
N4-acetylcytidine	Nucleotide	Pyrimidine Metabolism, Cytidine containing	-20.2 (-26.1, -14.0)	6.2E-06
6-bromotryptophan	Amino Acid	Tryptophan Metabolism	-18.6 (-23.3, -13.5)	4.5E-08
Nervonoylcarnitine (C24:1)*	Lipid	Fatty Acid Metabolism (Acyl Carnitine, Monounsaturated)	-18.0 (-23.2, -12.4)	4.5E-06
Kynurenine	Amino Acid	Tryptophan Metabolism	-14.8 (-19.1, -10.3)	1.9E-06
*Higher in non-Hispanic Black women*				
Thymol sulfate	Xenobiotics	Food Component/Plant	155.6 (86.2, 250.9)	8.1E-06
2-naphthol sulfate	Xenobiotics	Chemical	95.5 (58.1, 141.7)	8.1E-07
2-hydroxyfluorene sulfate	Xenobiotics	Tobacco Metabolite	77.1 (50.9, 107.7)	4.6E-09
Carotene diol (3)	Cofactors and Vitamins	Vitamin A Metabolism	37.4 (24.7, 51.4)	2.2E-07
N-acetylarginine	Amino Acid	Urea cycle; Arginine and Proline Metabolism	33.3 (22.0, 45.6)	3.0E-07
N-acetylglutamine	Amino Acid	Glutamate Metabolism	26.1 (17.7, 35.0)	6.8E-08
3-methoxytyrosine	Amino Acid	Tyrosine Metabolism	25.2 (17.7, 33.1)	1.8E-09

^a^Multivariable linear regression analysis on the log-transformed metabolites and race (non-Hispanic white, non-Hispanic black) difference was performed, adjusted for age, body mass index (BMI), alcohol consumption, and education

^b^Multiple hypothesis testing was accounted for by implementing the Bonferroni correction. Statistical significance was defined as a Bonferroni-adjusted *p*-value <

^c^Percentage difference was back-transformed linear regression coefficients, calculated as ${100\times (e}^{\beta }-1)$, with a 95% confidence interval. A negative percentage difference indicates that the metabolite is lower in NHB women compared with NHW women

^d^Asteriks in the metabolite biochemical name indicates that the metabolite has not been confirmed based on a standard, but we are confident in its identity (not Metabolomics Standards Initiative Tier 1 identification)

### Percentage differences in metabolites by race

Seven metabolites belonging to xenobiotics (*n* = 5) and amino acid (*n* = 2) super-pathways exhibited an absolute percentage difference ≥ 50% between NHB and NHW women. Further, 10, 17, and 23 metabolites exhibited absolute percentage differences ≥ 40%, ≥ 30%, and ≥ 20%, respectively. Table 4 summarizes the number of metabolites showing ≥ 20% absolute percentage difference between NHB and NHW women and provides an overview of the differences. Most differences were in xenobiotics super-pathway, particularly in xanthine metabolism (2 lower in NHBs), chemicals (3 lower and 1 higher in NHBs), and tobacco metabolites (1 higher in NHBs). In the amino acid super-pathway, we observed several differences across different sub-pathways including lysine metabolism (2 lower in NHBs), leucine, isoleucine, and valine metabolism (3 lower in NHBs), urea cycle, arginine and proline metabolism (1 higher in NHBs). We also observed differences in lipid metabolism (sphingosines), cofactors and vitamins (vitamin A metabolism), and nucleotide metabolism.

**Table 4:** Metabolites with ≥ 20% absolute percentage difference between non-Hispanic Black (NHB) and non-Hispanic White (NHW) women^a, b, c^

Super-pathway^d^	Sub-pathway	No of Metabolites	Direction of Effect^e^	
			(↓) in NHB	(↑) in NHB
Xenobiotics	Xanthine Metabolism	9	2	0
	Tobacco Metabolite		0	1
	Food Component/Plant		0	1
	Chemical		3	1
	Bacterial/Fungal		1	0
Partially Characterized Molecules	Partially Characterized Molecules	1	1	0
Nucleotide		1	1	0
Lipid	Sphingosines	1	1	0
Cofactors and Vitamins	Vitamin A Metabolism	1	0	1
Amino Acid	Urea cycle; Arginine and Proline Metabolism	10	0	1
	Tyrosine Metabolism		0	1
	Tryptophan Metabolism		1	0
	Lysine Metabolism		2	0
	Leucine, Isoleucine and Valine Metabolism		3	0
	Alanine and Aspartate Metabolism		1	0
	Glutamate Metabolism		0	1

^a^Multivariable linear regression analysis on the log-transformed metabolites and race (NHB vs NHW), adjusting for age, BMI at enrollment, alcohol consumption, and education

^b^Multiple hypothesis testing was accounted for by Bonferroni correction. Statistical significance was defined with a Bonferroni-adjusted$p-\text{value}<{10}^{-5}$

^c^Percentage difference was back-transformed linear regression coefficients, calculated as $100\times \left({e}^{\beta }-1\right)$. Metabolites with an absolute percentage difference of 20% and above were listed

^d^Carbohydrate, energy, and peptide super-pathways did not contain any metabolite with a significant difference (≥ 20%) between groups and are therefore not included in this table

^e^Direction of effect indicated lower (↓) or higher (↑) metabolite in NHB compared with NHW women. The number represented the metabolites count within each sub-pathway

The top 5 metabolites with the highest percentage differences in NHB women compared to NHW women were thymol sulfate (155.6%$,{p=8.1\times}{10}^{-6}$)[food component/plant], 2-naphthol sulfate (95.5%$,{p=8.1\times}{10}^{-7}$)[chemical], 2-hydroxyfluorene sulfate (77.1%$,{p=4.6\times}{10}^{-9}$)[tobacco metabolite], carotene diol (3) (37.4%$,{p =2.2\times}{10}^{-7}$)[vitamin A metabolism], and N-acetylarginine (33.3%, *p* = 3.0${\times}{10}^{-7}$)[urea cycle, arginine and proline metabolism]. Conversely, the top 5 metabolites with the highest percentage differences in NHW women than NHB women were N6-methyllysine (60.5%$,{p=6.7\times}{10}^{-29}$)[lysine metabolism], tryptophan betaine (58.4%, *p* = ${9.2\times}{10}^{-12}$)[tryptophan metabolism], theophylline (52.7%$,{p=3.1\times}{10}^{-7}$)[xanthine metabolism], N-methylpipecolate (50.4%$,{p=1.4 \times}{10}^{-14}$)[bacterial/fungal], and metabolonic lactone sulfate (48.7%$,{p=1.6 \times}{10}^{-15}$)[partially characterized molecules].

The results from the sensitivity analysis limited to women without a family history of breast cancer (*N* = 516) were largely consistent with the overall analysis. Five metabolites including carotene diol (3), 6-bromotryptophan, perfluorohexanesulfonate (PFHxS), aspartate, and thymol sulfate were associated with race in the overall analysis but were not statistically significant in the sensitivity analysis (Additional file 1: Table S3). However, the directions and magnitude of associations remained the same (Table 3; Additional file 1: Table S3).

## Discussion

Our study of 163 NHB and 506 NHW women, using untargeted metabolomics and analyzing 828 metabolites, offers a comprehensive perspective on racial differences in metabolomic profiles between NHB and NHW women. At the super-pathway level, we found significant differences in several xenobiotics and amino acid metabolites. At the sub-pathway level, we found higher levels of metabolites of tobacco in NHB women and higher levels of xanthine metabolites as well as lysine and tryptophan metabolites in NHW women. When compared to recent studies, our findings both corroborate existing knowledge and extend our understanding of racial metabolomic differences.

We found strong correlations of BMI with several metabolites, particularly in lipid and amino acid metabolism super-pathways. Importantly, racial differences in metabolic profiles persisted after adjusting for BMI. This indicates that the observed differences in metabolites between NHB and NHW women cannot be attributed to differences in BMI. This finding is crucial, given the differences in BMI between NHB and NHW women [24]. Our results suggest that other biological factors likely play a role in these metabolic differences.

NHB women had higher levels of certain xenobiotics, suggesting differences in environmental exposures [25]. Among the xenobiotics, thymol sulfate, 2-naphthol sulfate, and 2-hydroxyfluorene sulfate were highest in NHB women. These metabolites are derived from exposure to polycyclic aromatic hydrocarbons (PAHs) found in air pollution, cigarette smoke, and grilled foods [26, 27]. Socioeconomic factors, residential location, smoking habits, and secondhand smoke exposure might explain these differences [28]. These differences can be linked to a higher risk of smoking-related diseases like lung cancer that disproportionately affect NHB populations [29]. It is also important to consider the potential role of genetic polymorphisms in xenobiotic metabolizing enzymes [30]. Metabolism and excretion of xenobiotics are different between NHB and NHW women [31]. Therefore, differences in enzyme activity could contribute to the observed differences in xenobiotic metabolites between NHB and NHW women. Higher levels of theophylline and 1,7-dimethylurate within the xanthine metabolism sub-pathway in NHW women suggest potential differences in caffeine consumption or metabolism, aligning with previous findings of higher caffeine intake among NHW individuals [32].

NHB women had higher levels of N-acetylarginine, N-acetylcitrulline, and trans-4-hydroxyproline (the latter two being marginally significant after Bonferroni correction) which are part of the urea cycle, arginine, and proline metabolism. These acetylated amino acids are associated with kidney function [33], and their higher levels in NHB women may serve as potential markers of differences in kidney function between NHB and NHW women. While these differences could contribute to observed racial differences in kidney diseases, it's important to note that they might also be indicators of underlying physiological differences or responses to environmental factors. Conversely, we observed lower levels of tryptophan betaine, 6-bromotryptophan, and kynurenine, all belonging to the tryptophan metabolism sub-pathway, in NHB women. These differences in tryptophan sub-pathway metabolites could reflect differences in tryptophan metabolism between NHB and NHW women, potentially serving as indicators of metabolic differences that may influence disease risk. Tryptophan metabolism plays a role in different physiological processes, including immune regulation and neuronal function, and has been associated with several diseases. For instance, recent research on the tryptophan-kynurenine pathway in colorectal cancer patients has found that higher tryptophan is associated with lower mortality risk [34]. Given our observation of lower tryptophan metabolites in NHB women, this might point to racial differences in cancer outcomes, although further research is needed to establish any casual associations. Tryptophan metabolism has also been increasingly linked to Alzheimer’s disease [35]. Lower 6-bromotryptophan levels have been associated with a higher risk of chronic kidney disease progression in NHBs [36], but whether this is a causal factor or a marker of disease progression requires further investigation. Lower tryptophan betaine in NHBs could reflect dietary differences as this metabolite is a marker of legume and nuts intake [37]. While lower tryptophan betaine levels have been associated with endothelial dysfunction and inflammation, which are known risk factors for cardiovascular disease [38, 39], it's crucial to consider that these associations may be complex and multifactorial. Metabolites from the lysine metabolism sub-pathway including N6-methyllysine and N6,N6-dimethyllysine showed a similar association. While lysine methylation is linked to cancer progression [40], these metabolic differences should be interpreted cautiously. They may serve as potential biomarkers for differences in cellular processes between NHB and NHW women, but their direct role in disease causation or progression needs further investigation.

Our study found lower levels of hexadecasphingosine (sphingosines sub-pathway) and nervonoylcarnitine (fatty acid metabolism sub-pathway) in NHB women, both belonging to the lipid super-pathway. These findings provide insights into potential racial differences in lipid metabolism. Hexadecasphingosine is a component of sphingomyelin, which itself has been associated with cardiovascular diseases [41]. It's important to note that our current study focused on metabolites and included only 305 lipids, which represents a fraction of the lipidome. Our observations align with a separate, more comprehensive lipidomics study we conducted, which demonstrated overall lower lipid species levels, particularly triacylglycerols, in NHB women [42]. These differences could reflect different dietary patterns, metabolic efficiency, or other environmental factors, and may not necessarily indicate a predisposition to specific health outcomes. Further research is needed to understand the functional significance of these lipid and metabolite differences between NHB and NHW women.

A previous study from the Women’s Health Initiative-Observational Study; 138 NHB and 696 NHW women found differences in metabolites between NHB and NHWwomen. The researchers identified a group of 152 metabolites out of the 472 metabolites they analyzed, especially lipid metabolites and amino acids, that were significantly different between NHB and NHW women [14]. Our study used an untargeted approach and we analyzed more metabolites (828 vs 472), which provided us with the opportunity to identify more metabolites that may differ between NHB and NHW women. For instance, we also identified significant differences in specific xenobiotic metabolites (e.g., thymol sulfate, 2-naphthol sulfate) that were not identified in their study, potentially indicating important racial differences in environmental exposures or metabolism of exogenous compounds. This highlights the importance of comprehensive metabolite profiling in capturing the full spectrum of racial metabolomic differences. This broader coverage allows for a more comprehensive assessment of racial differences in metabolic profiles. Another study performed a metabolomic analysis in 175 women and 175 men, including 171 NHBs and 179 NHWs, and analyzed 892 metabolites [15]. They reported that the majority of differentially abundant metabolites were lower in NHB participants, particularly in lipid and amino acid metabolism. Our main findings align with theirs, but our study's larger sample size (669 vs. 175 women) and stringent Bonferroni correction for multiple comparisons enhance the reliability and statistical robustness of our findings. While we identified fewer differential metabolites (26 out of 828) compared to both prior studies, our findings are less likely to be due to chance or Type I error because of our stringent correction for multiple testing. The consistency in some findings (e.g., lower levels of N6-methyllysine and tryptophan betaine in NHBs, higher levels of N-acetylarginine in NHBs) across studies, despite different methodologies, strengthens the evidence for these particular racial differences.

Our study has several strengths. Our study population is fairly large with a sizeable proportion of NHB women, allowing robust analysis of racial differences. We accounted for potential confounders by adjusting our models for age, BMI, alcohol consumption, and education. Alcohol intake is known to influence metabolic profiles. Education was included in the model to account for the potential influence of socioeconomic status on the observed associations. BMI is correlated with many of the metabolites. We had detailed information on BMI collected on the same day of plasma sample procurement, which enabled us to perform the most accurate adjustment for this variable in our analysis. However, our study had some limitations. We included only premenopausal women and our findings may not apply to men. Our study is observational and the biological significance of several observed differences requires evaluation and validation in other studies. We lacked data on diet, tobacco use, and comorbidities, which may influence metabolic profiles and potentially confound the observed associations. Additionally, like all observational studies, residual confounding could still be an issue, and our findings should be interpreted within that context. Future studies should investigate the underlying genetic and environmental factors contributing to these metabolomic differences and confirm whether these observed differences contribute to racial disparities in health outcomes and disease susceptibility across diverse populations.

## Conclusions

We report differences in several metabolites between NHW and NHB women. These findings require validation in a different study and could provide insight into investigating how racial differences in metabolites may impact disease burden across diverse populations.

## Supplementary Information

Additional file 1: Tables S1–S3. Table S1: Table S1. Correlations of metabolites and body mass index (BMI) showing absolute correlation coefficients ≥0.3. Table S2. List of all metabolites analyzed in association with race (non-Hispanic Black vs. non-Hispanic White women). Table S3. Metabolites with significant differences between non-Hispanic Black and non-Hispanic White women without a family history of breast cancer (*N*=516).

## Data Availability

Data described in the manuscript, code book, and analysis code will be made available upon request and approval.

## Abbreviations

NHW: Non-Hispanic White
NHB: Non-Hispanic Black
BMI: Body mass index
